# Putting Actions in Context: Visual Action Adaptation Aftereffects Are Modulated by Social Contexts

**DOI:** 10.1371/journal.pone.0086502

**Published:** 2014-01-22

**Authors:** Stephan de la Rosa, Stephan Streuber, Martin Giese, Heinrich H. Bülthoff, Cristóbal Curio

**Affiliations:** 1 Max Planck Institute for Biological Cybernetics, Tübingen, Germany; 2 Hertie Institute for Clinical Brain Research at Tübingen University, Tübingen, Germany; 3 Department of Brain and Cognitive Engineering, College of Information and Communication, Korea University, Seol, Korea; VU University Amsterdam, Netherlands

## Abstract

The social context in which an action is embedded provides important information for the interpretation of an action. Is this social context integrated during the visual recognition of an action? We used a behavioural visual adaptation paradigm to address this question and measured participants’ perceptual bias of a test action after they were adapted to one of two adaptors (adaptation after-effect). The action adaptation after-effect was measured for the same set of adaptors in two different social contexts. Our results indicate that the size of the adaptation effect varied with social context (social context modulation) although the physical appearance of the adaptors remained unchanged. Three additional experiments provided evidence that the observed social context modulation of the adaptation effect are owed to the adaptation of visual action recognition processes. We found that adaptation is critical for the social context modulation (experiment 2). Moreover, the effect is not mediated by emotional content of the action alone (experiment 3) and visual information about the action seems to be critical for the emergence of action adaptation effects (experiment 4). Taken together these results suggest that processes underlying visual action recognition are sensitive to the social context of an action.

## Introduction

Actions rarely come out of the blue but are typically embedded in an action sequence (*social context*). The social context provides important information about the social meaning of an action. For example, laughing after having someone mislead in a prank is considered to be ‘laughing about someone’ while laughing with someone about a joke is often referred to as ‘laughing with someone’. These examples point to the importance of integrating an action into its social context to properly understand someone else’s action. The psychological mechanisms contributing to context sensitive action recognition are poorly understood.

A substantial amount of action recognition research has focused on the investigation of isolated actions, i.e. actions that were not embedded in an social context. This important research has demonstrated several key aspects of action recognition. For example, visual action recognition is sensitive to high level visual features [1], motor action expertise [2], action execution [3], and motor training [4]. These findings can be described within physiologically plausible computational models of visual action recognition [5]–[7]. These models suggest that many of the observed effects in action recognition can be explained by feed-forward processing of visual information. However, other factors, such as social context, have received little attention in previous research.

Embedding actions into their social context is important for understanding the social intention of an action [8]. Jacob and Jeannerod (2005) highlighted this important aspect of action understanding by employing the example of Dr. Jekyll and Mr. Hyde. Being the same physical person in two different cognitive states, Dr. Jekyll and Mr. Hyde follow different surgical goals on humans. Dr. Jekyll carries out surgeries on anesthetized patients for the sake of healing. Mr. Hyde also carries out surgeries but on non-anesthetized patients for all the wrong reasons. If someone were to simply observe the physical action patterns of Dr. Jekyll and Mr. Hyde, one would not be able grasp the difference regarding the action intention between these two situations. Only knowing whether Dr. Jekyll transformed into Mr. Hyde would allow a full understanding of Mr. Hyde’s actions. Hence, the integration of actions into their context allows one to fully understand the action including its intention. Examining how humans integrate visual action information into their social context furthers our understanding of how humans understand social intentions. Specifically, it provides insights about whether visual action recognition mechanisms are sensitive to the semantic (e.g. intentional) aspects of an action. Thereby, this research contributes to the ongoing debate about which aspects of an action (e.g. physical properties or action goals) are recognized during the recognition of an action [8], [9].

So far, previous research showed that the visual observation of an interaction partner in an interactive table tennis task depends on the nature of the interaction (cooperative or competitive play) [10]. Moreover fMRI studies provide some evidence that concurrently presented object context, but not social context, modulates the BOLD response in the inferior frontal gyrus [11] - a cortical area considered to be critical for action understanding. However, at least part of this modulation is owed to physical properties of the observed action rather than the action intention implied by the object context [12]. Overall, this research provides only limited insights into how visual action information and the social context are integrated and leaves the question unanswered, which action recognition processes are sensitive to social context. One possible way in which social context can exert influence on action recognition is by affecting action recognition after visual information about an action has been mapped onto semantic action knowledge. In this case, social context should influence mainly non-visual processes about an action. Alternatively, action recognition could affect visual action recognition processes concerned with the processing of visual action information (e.g. action information from body postures). In the current study we set out to probe the influence on social context on action adaptation using visual and non-visual action information to further our understanding about how social context is integrated within the action recognition hierarchy.

We used a visual adaptation paradigm to examine the effect of social context on action recognition. Adaptation is a widely used paradigm to behaviorally probe visual recognition processes [13], [14]. Because adaptation is believed to have the ability to target specific aspects of perceptual-cognitive processes, adaptation paradigms have also been termed the “psychophysicist electrode”. In a visual adaptation paradigm, participants view an (adaptor) stimulus for a prolonged amount of time and subsequently report their perception of an ambiguous looking test stimulus. A typical finding is that the perception of the ambiguous test stimulus is biased away from the adapted stimulus. For example, adapting to a left tilted line will bias participants to report a vertical presented line oriented to the right [15]. Adaptation effects have been typically taken as evidence for the sensitivity of the underlying recognition processes to the manipulated visual properties (e.g. in the previous example: orientation sensitivity). In this way, adaptation paradigms have been commonly used to draw inferences about the tuning properties of neural processes underlying visual recognition [16], [17]. With regards to action recognition, the results of action adaptation paradigms have been shown to be in line with the results obtained from physiological recordings from cortical units sensitive to actions [18]. Here we employ an adaptation paradigm to examine the sensitivity of action recognition processes to social contexts.

Previous studies employed visual adaptation paradigms to examine mechanisms underlying the recognition of object-directed [18] and locomotive actions [1]. Here, we examined for the first time action recognition mechanisms involved in the recognition of different social action categories (e.g. hitting or shaking hands) and their sensitivity to social context. Specifically, we investigated visual adaptation to different social actions to examine the sensitivity of action recognition processes to social contexts. We reasoned that if social contexts directly influence mechanisms underlying action recognition, then action adaptation after-effects should be modulated by social contexts (experiment 1) and visual action information should be important for the the emergence of this effect (experiment 2–4).

## Experiment 1

We examined the effect of social context on action recognition by creating two action adaptation conditions. In brief, the two experimental conditions had identical adaptor and test images and differed with respect to the *social context* that was provided prior to the presentation of one of the adaptors. A modulation of the action adaptation effect across these two experimental conditions would indicate that social context influences the adaptation effect and thereby also the visual action recognition processes underlying the adaptation effect.

**Figure 1:**
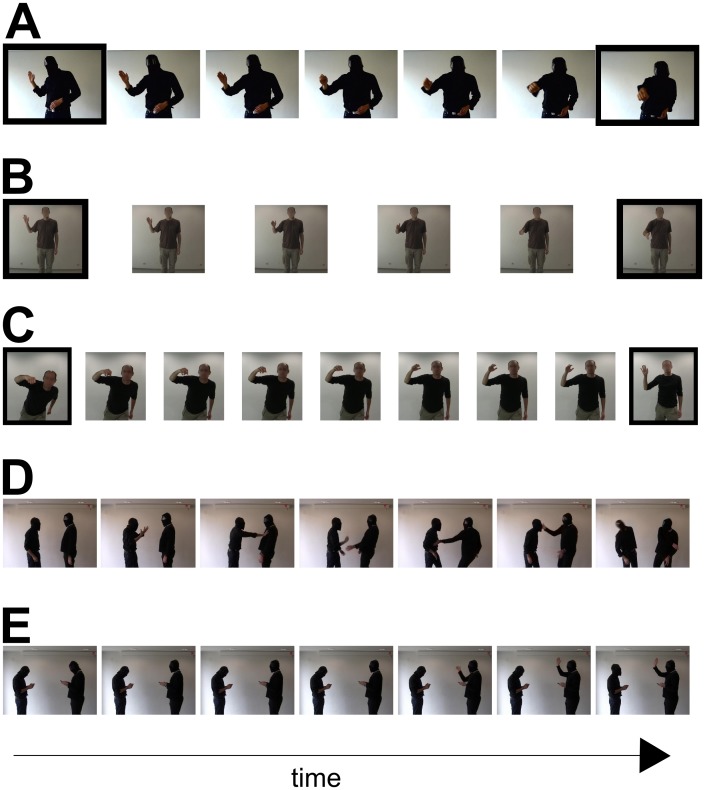
Example stimuli. **A.** Test stimuli of experiment 1 and 2. The images with the black borders also served as adaptors in experiment 1. **B**. Test stimuli of the same emotion condition of experiment 3. Black borders indicate the images that were used as adaptor stimuli. **C**. Test stimuli of the different emotion condition experiment 3 and experiment 4. The images with the black borders served as adaptors in experiment 3 only. **D.** Stills of the hitting video. The left person pushed the right person (stills 1 to 4), who in return slapped the left person (stills 5–7). Still 5 shows the apex of the swing before the actual slap of the right person. **E.** Stills of the the waving video. The left person is looking up (still 2) and starts waving at the right person (stills 5–7). The time line only applies to panel D and E.

Both experimental conditions probed adaptation by presenting two types of adaptors. One adaptor showed an image of a person at the apex of a fist punch (hitting adaptor) and the other adaptor showed an image of a person holding up a hand (hand-up adaptor) (see Figure 1). Importantly, the interpretation of the hand-up adaptor was ambiguous as suggested by a pilot experiment, which showed that about half of the observers interpreted the hand-up adaptor as a person waving and remaining observers as a person taking a swing for a hit. This ambiguous hand-up adaptor was critical for the assessment of the social context sensitivity of visual action recognition processes as explained in the following.

We manipulated the social context preceding this ambiguous hand-up adaptor across the two experimental conditions. The context manipulation consisted of the presentation of a video right before the hand-up adaptor (no video was shown prior to the presentation of the hitting adaptor). In one experimental condition (hitting context condition) the hand-up adaptor was preceded by a video of one person hitting another one; in the other experimental condition (waving context condition) the hand-up adaptor was preceded by a video showing one person waving at another one. We expected the context videos to only prime the action interpretation of the ambiguous hand-up adaptor. In particular, we expected that the hitting video would bias participants to interpret the hand-up adaptor in the hitting context condition as ‘taking a swing for a hit’. As a result, participants would interpret both the hitting and the hand-up adaptor in the hitting context condition in a congruent fashion, namely as ‘hitting’. In contrast, we expected that the waving video would cause participants to perceive the hand-up adaptor as waving. Consequently, participants’ interpretation of the hitting and the hand-up adaptor in the waving context condition should be incongruent: the hand-up adaptor should be perceived as a waving action and the hitting adaptor should be perceived as a hitting action.

We reasoned that if action recognition is sensitive to social context then action adaptation should be modulated by the change of the hand-up adaptor’s action interpretation between the hitting and the waving context condition. Specifically, in the hitting context condition, both the hitting and the hand-up adaptor should be interpreted as the same or very similar action (i.e. hitting). Adapting to the same action should lead to the same response bias and therefore to no or a small adaptation effect. In contrast, in the waving context condition both the hitting and the hand-up adaptor should be associated with incongruent action interpretations (hitting and waving, respectively). Adapting to different actions should result in different response biases and consequently in the emergence of an action adaptation effect.

In summary, we expected a modulation of the adaptation after-effect between the hitting and waving context condition, namely we anticipated a smaller adaptation after-effect in the hitting compared to the waving condition if social context modulates the action adaptation effect.

### Methods

#### Participants

15 participants participated in the hitting context experiment (7 females, mean age = 27.4, SD = 5.31) and another 15 participants in the waving context experiment (9 females; mean age = 26.0; SD = 5.39). All participants were naïve to the stimuli and the experimental procedure. Participants gave written informed consent for their participation prior to the experiment. The experiment was conducted in line with the Declaration of Helsinki and approved by the ethics board of the Max Planck Society (Ethikrat).

#### Stimuli and Apparatus

Test and adaptor images were rendered from a video showing a person changing his body posture from a hand-up posture to a hitting posture (Figure 1A). In total seven frames were rendered from this video. The first frame (showing a static hand-up posture) and the last frame (showing a static hitting posture) served as adaptor stimuli. All seven stimuli in Figure 1A were used as test stimuli. Note, that both test and adaptor stimuli showed static images of actions from a second person perspective (i.e. from the perspective of the interaction partner). In addition to the test and adaptor stimuli, we presented two videos of the same length (2 s) showing two persons interacting with each other (Figure 1D & E). In one video one man was hitting another one (hitting context video) (Figure 1D). In the other video, one man waved at another one (waving context video) (Figure 1E). These images meant to provide the social context for the hand-up adaptor. To ensure that the entire social context is visible (i.e. both actors) we recorded the social context videos from a third person perspective. We introduced a 820 ms blank screen between the presentation of the video and the onset of the adaptor image to increase the likelihood that observers felt the action displayed in the adaptor image to be a reaction to the social context shown in the video. This blank interval time was chosen based on subjective assessment of the image material by one of the authors and a graduate from a film academy. Longer blank intervals gave rise to the impression that the adaptor image was unrelated to the social context. Shorter blank intervals led to the impression that the adaptor image action was not a plausible consequence of the social context (e.g. it felt there was too little time for a person to be able to lift his hand within a shorter amount of time).

In all instances the face of the person was masked or blurred to minimize the potential effects of facial expressions on action adaptation. Stimuli were presented on a LCD monitor (refresh rate of 60 Hz; screen resolution of 1280×1024 pixels) using Matlab and the Psychophysics Toolbox 3 [19]–[21]. The stimulus recording was done using a Canon HF100 camcorder with 60 fps.

#### Procedure

At the beginning of the experiment we informed participants about the two actions of experiment 1. Specifically, we told participants that they were going to see two actions, namely “action 1” and “action 2”. This instruction meant to avoid biasing participants’ action interpretation. To give concrete examples for “action 1” and “action 2”, we showed images of “action 1” and “action 2” visually. Specifically, we showed the hitting adaptor (∼4 s presentation time) for “action 1” and the hitting or waving video (depending on the condition) followed by the hand-up adaptor for “action 2”. Participants were asked to remember which action was associated with which visual image. At not point we provided semantic interpretations of the displayed actions.

**Figure 2:**
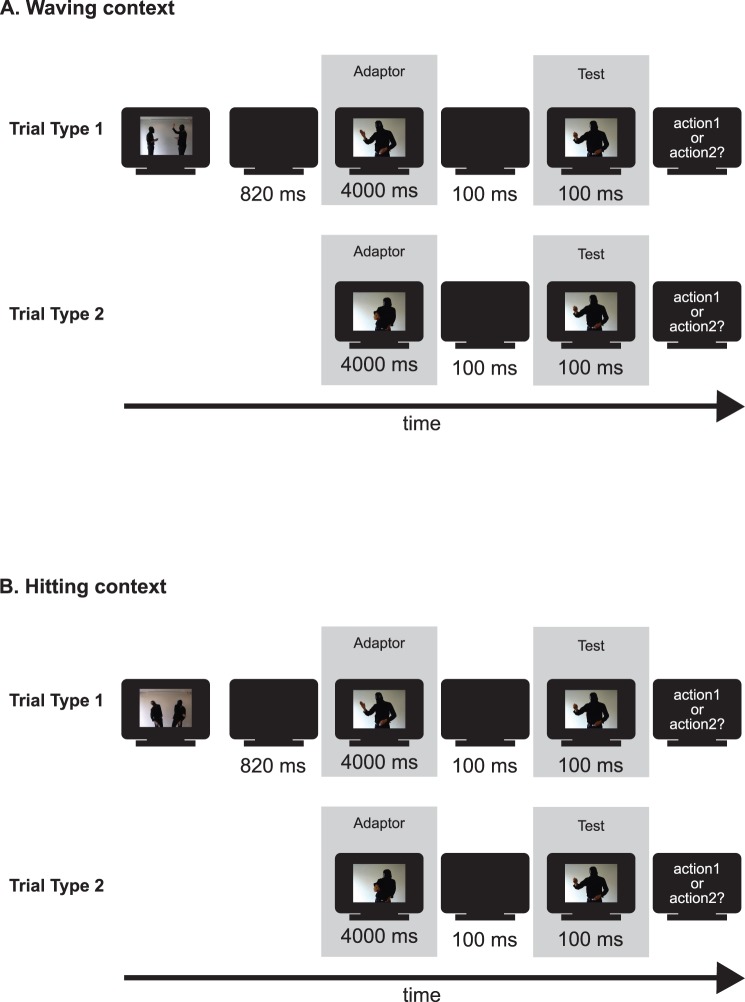
Experimental procedure. Schematic representation of the stimulus presentation procedure of the waving context condition (**A**) and the hitting context condition (**B**) of experiment 1. Each experimental conditions consists of two types of trials (shown at the top and bottom of each panel). The video displayed in Figure 1E was shown first in the first type of trial of the waving condition. The video displayed in Figure 1D was shown first in the first type of trial of the hitting condition. The presentation durations for each screen are given below each screen.

The waving context condition consisted of two types of experimental trials (Figure 2A). The first type of trial consisted of the presentation of a video showing one person waving at another one (2400 ms), a black screen (820 ms), the hand-up adaptor (4000 ms), a black screen (100 ms), a test stimulus (100 ms), and the not time-restricted answer interval. The second trial type consisted of the presentation of the hitting adaptor (4000 ms), a black screen (100 ms), a test stimulus (100 ms), and the non time-restricted answer interval.

The hitting context condition also consisted of the following two types of experimental trials (Figure 2B). The first type of trial consisted of the presentation of a video showing one person hitting another one (2400 ms), a black screen (820 ms), the hand-up adaptor (4000 ms), a black screen (100 ms), a test stimulus (100 ms), and the not time-restricted answer interval. The second trial type consisted of the presentation of the hitting adaptor (4000 ms), a black screen (100 ms), a test stimulus (100 ms), and the non time-restricted answer interval.

Participants’ task was to report their perception of the test stimulus as either ‘action 1’ or ‘action 2’ (in accordance to their specific instructions) by pressing one of two buttons on the keyboard (‘z’ and ‘<’ on an English keyboard layout). To inform participants about the action-answer key mapping, participants were shown an adaptor image (printed on paper) that was associated with each answer key in the instruction phase of the experiment. The action-keyboard assignment was counterbalanced across participants. Participants received 20 practice trials in which participants could practice and resolve any outstanding question regarding the task. The practice trials showed that participants understood the task. The data of the practice trials were discarded from the analysis. The experiment took about 1.5–2 hours.

#### Analysis and Design

Psychometric functions were used to describe the relationship between physical appearance of the test stimuli and perceived appearance. Psychometric functions were fitted using a Weibull function [22] with α (position of the psychometric function along on the x-axis) and β (slope of the psychometric function), and λ (lapse rate) as free parameters (gamma was fixed to 0). The fits were done for each experimental condition, adaptor, and participant separately. Action adaptation after-effects were measured as the difference in perception of a test stimulus between trials showing the hand-up adaptor and trials showing the hitting adaptor. Specifically we measured the shift of the psychometric functions at the point of subjective equality (PSE) (i.e. the stimulus level that produced 50% of action 1 responses and 50% of action 2 responses).

When fitting psychometric functions to the data, no significant differences with respect to β were found (detailed results not reported here). Moreover, using the method of constant stimuli each of the 9 test stimuli was shown in each adaptor condition 15 times. The presentation order of the test and adaptor stimuli was randomized.

### Results

**Figure 3:**
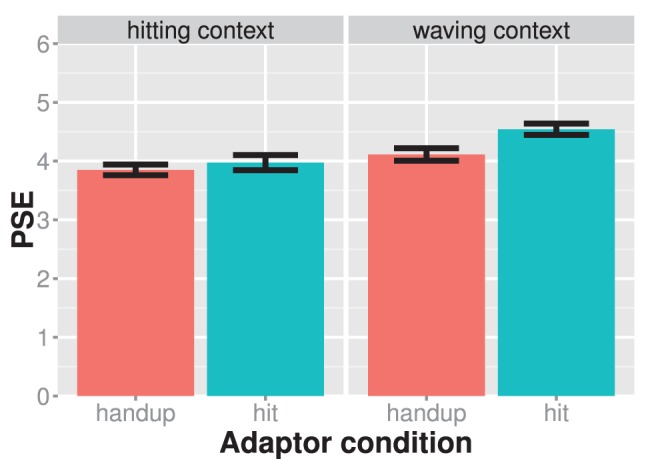
Results of experiment 1. The mean PSE values are shown for each experiment condition (different panels) and adaptor condition separately. The left panel shows the hitting context condition and the right panel shows the waving context condition. Larger PSE values indicate that the test stimulus showed more of a hitting action. Adaptation effects are assessed in a similar vein here and in all subsequent figures. Significant PSE differences between adaptor conditions of the same experiment condition are indicative of an adaptation effect. Bars indicate one standard error of the mean.

The shift of the psychometric functions between the hand-up and hit adaptor appeared larger in the waving than in the hitting context condition (Figure 3). A mixed ANOVA with adaptor condition as a within, context as a between-subject factor, and mean PSEs as the dependent variable showed a significant main effect of context, F(1,28) = 9.17; η^2^_partial_ = 0.24; p = 0.005, and adaptor, F(1,28) = 19.94; η^2^_partial_ = 0.42; p<0.001. Importantly, the interaction between adaptor and context was significant, F(1,28) = 6.08; η^2^_partial_ = 0.18; p = 0.020, suggesting that the size of the adaptation after-effect was modulated by social context. In line with our prediction, the adaptation effect was larger in the waving, M = 0.45, than in the hitting condition, M = 0.11. Hence, social context seems to modulate action adaptation after-effects.

In experiment 1 participants’ response were biased opposite to the adapting stimuli. Although such repelling effects are in line with previous reports about action adaptation effects [23], they are not unique to action adaptation paradigms. For example, similar effects are expected from reverse priming effects [24]. One major difference between these alternative explanations and adaptation is that they often require a much shorter presentation time (i.e. no long adaptation phase) in order to effectively bias participants responses. In experiment 2, we wanted to ensure that the social context modulation of the adaptation effect is owed to the adaptation phase. We therefore removed the adaptation phase from experiment 1 and tested the action adaptation effect.

## Experiment 2

To ensure that the observed social context modulation of action adaptation is owed to adaptation, we removed the adaptation phase from the waving context condition of experiment 1. We expected that if the adaptation phase is not required for observed effects in experiment 1, the presentation of the social context alone should be sufficient to induce a social context modulation of the adaptation effect. To this end, we removed the presentation of the hand-up adaptor from the waving condition of experiment 1 resulting in the presentation of the waving video only. Participants adapted to this waving video and the hitting adaptor in experiment 2. To assess whether the modulation of the adaptation effect occurred despite removing the hand-up adaptor, we compared the PSEs of experiment 2 with those of the hitting condition of experiment 1.

### Methods

The methods for experiment 2 were identical to those of experiment 1 with the following exceptions.

#### Participants

15 participants participated in the experiment (6 females, mean age = 26.43, SD = 2.99). All participants were naïve to the stimuli and the experimental procedure. None of the participants participated in the previous experiments of the study.

#### Stimuli and Apparatus

The same stimuli as in experiment 1 were used.

#### Procedure

Experiment 2 replicated the waving context condition of experiment 1 with the only difference that only the video (i.e. no hand-up adaptor) was presented as an adaptor (one time presentation). The experiment took about 1–1.25 hours.

### Results

**Figure 4:**
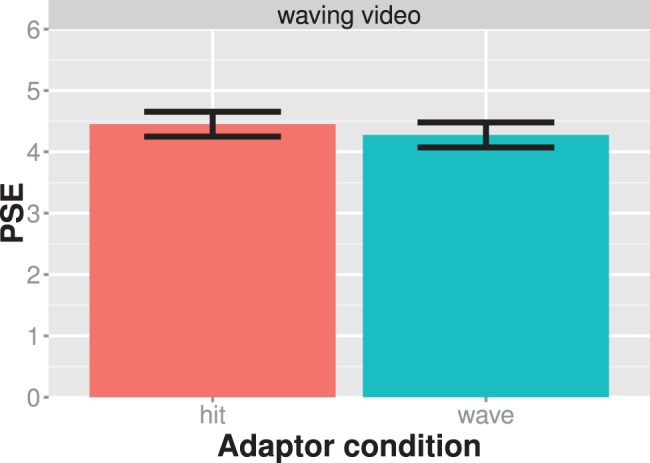
Results of experiment 2. The average PSE is shown for each adaptor condition separately. Larger PSE values indicate that the test stimulus showed more of a hitting action. Bars always indicate one standard error of the mean.

We compared the PSEs of experiment 2 (Figure 4) with those of the hitting context condition of experiment 1 (Figure 3 left panel). This comparison showed a significant between-subject main effect of condition, F(1,26) = 6.319; η^2^_partial_ = 0.20; p = 0.0185, a significant within-subject main effect of adaptor, F(1,26) = 6.514; η^2^_partial_ = 0.20; p = 0.0169, but no significant interaction between condition and adaptor, F(1,26) = 0.547; η^2^_partial_ = 0.02; p = 0.4461. The significant main effect of adaptor in the presence of a non-significant interaction suggests that the presentation of the waving video caused an adaptation effect but it was unable induce a social context modulation of the adaptation after-effect. The lack of an interaction effect is only indirect evidence for the adaptor image being important for the social context modulation of the adaptation effect.

We attempted to find more direct evidence for the adaptor phase being important for action adaptation by comparing the adaptation effect of video alone condition of experiment 2 and with the waving context condition (i.e. video plus adaptor phase) of experiment 1. We compared the two conditions in a two way mixed ANOVA with adaptor as a within subject factor and experiment as a between subject factor. We expected the interaction between adaptor and experiment to be significant if the presentation of the adaptor phase is important for action adaptation. Our results showed a non-significant main effect of experiment, F(1,28) = 0.51; η^2^_partial_ <0.01; p = 0.86, a significant main effect of adaptor, F(1,28) = 24.04, η^2^_partial_ = 0.47; p<0.001, and a significant interaction between adaptor and experiment, F(1,28) = 3.91, η^2^_partial_ = 0.13; p = 0.048. The significant interaction indicates that the adaptation effect was significantly larger in the waving context condition (M = 0.43) than in the waving video only condition (M = 0.18). In conclusion, the results of experiment 2 show the importance of the adaptor phase for action adaptation and for the social context modulation of the action adaptation effect.

## Experiment 3

An alternative explanation for the observed modulation of the adaptation effect in experiment 1 is the modulation of the adaptation effect by emotion. We addressed this alternative explanation in experiment 3. In experiment 1, participants might have adapted to the emotion displayed by the adaptor rather than to the displayed action. Specifically, the hitting context condition showed two adaptors with the same action interpretation and, hence, with similar emotions. In contrast, the waving condition showed two adaptors with different action interpretations which were possibly associated with different emotions. Because the congruency of the adaptors’ emotional content paralleled the congruency of the adaptors’ action interpretation across the hitting and waving condition in experiment 1, emotion adaptation could provide an alternative explanation for the modulation of the adaptation effect in experiment 1. In this case, the observed effects would not be indicative of social context sensitivity of action recognition processes but of emotion recognition processes. Here, we examined whether adaptation differences between the context conditions of experiment 1 were owed to emotion adaptation rather than action adaptation.

To address this question, we created two experimental conditions. One of the condition used two adaptors showing different actions and similar emotions (similar emotion condition). The other experimental condition consisted of two adaptors showing different actions and different emotions (different emotion condition). We expected that if emotions are critical for the adaptation effect, then the adaptation effect should be of different magnitude between the similar and different emotion condition.

In order to set up experimental conditions with similar and different emotional action content, we first assessed the emotional content of images showing a person in a hitting, waving, and handshake posture. We asked 14 participants to rate the emotional expression (angry, happy, disgusted, fearful, sad, surprised) of the hitting, waving, and handshake images on a 7-point scale (images were displayed on a computer screen). Bonferroni corrected paired-t-tests found that only waving was rated significantly happier than hitting, t_paired_ = 13.500; df = 13; Cohen’s d = 3.79; p<0.001, and hitting was rated significantly angrier than waving, t_paired_ = −14.170; df = 13; Cohen’s d = 3.61; p<0.001. The same comparison of handshake and waving ratings did not show any statistical significant differences across the six emotion ratings. We used these results to create the different and similar emotion condition. Specifically, in the different emotion condition, participants were adapted to two actions having different emotional content (i.e. waving and hitting). In contrast, in the same emotion condition, participants were adapted to two actions with similar emotional content (i.e. waving and handshake). If emotions are critical for the adaptation after-effects, we expect different magnitudes of the action adaptation effect between the similar and different emotion conditions.

### Methods

The methods for experiment 3 were similar to those of experiment 1 with the following exceptions.

#### Participants

5 participants participated in the similar emotional condition (7 females, mean age = 32.4, SD = 9.41) and another 15 participants participated in the different emotional condition (3 females; mean age = 31.8; SD = 11.86). All participants were naïve to the stimuli and the experimental procedure. None of the participants had participated in previous experiments of the study.

#### Stimuli and Apparatus

We rendered 6 frames from a person transitioning from a waving posture to a handshake posture to create static adaptor and test stimuli for the same emotion condition (Figure 1B). We rendered 9 frames from a video showing a person transitioning from a waving to a hitting posture to create the static adaptor and test stimuli for the different emotion condition (Figure 1C).

#### Procedure

An experimental trial consisted of the presentation of one of two adaptors (4000 ms), a black screen (100 ms), a test stimulus (100 ms), and the not time-restricted answer interval. In the same emotion condition the adaptors were an image of a person stretching out the hand for a handshake and an image of a person waving. In the different emotion condition one adaptor was an image of a person at the apex of a fist punch and the other adaptor was an image of a person waving. In experiments 3 we referred to the actions with their actual names when instructing participants. The experiment took about 1.5 hours.

### Results

**Figure 5:**
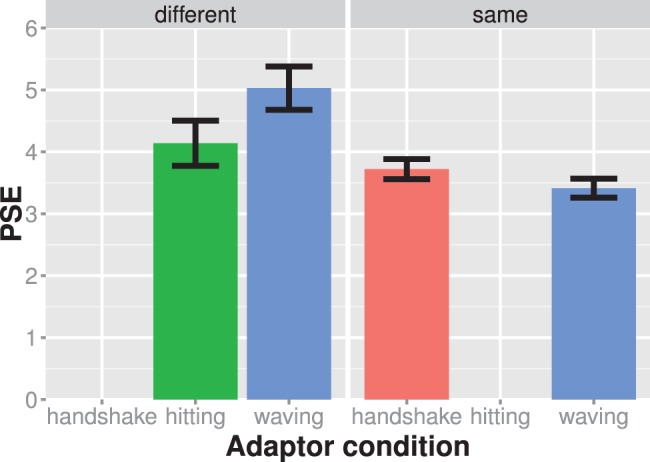
Results of experiment 3. The average PSE is shown for each experiment condition (different panels) and adaptor condition separately. The left panel shows the different emotion condition and the right panel shows the same emotion condition. Larger PSE values indicate that the test stimulus showed more of a waving action. Bars always indicate one standard error of the mean.

Figure 5 shows the results of this experiment. Both experimental condition seem to produce an adaptation effect. We assessed the adaptation effect difference between the same and different emotion condition using a mixed ANOVA with PSEs as a dependent variable, emotion condition (same vs. different) as a between subject factor and adaptor as a within subject factor. The main effect of emotion condition was significant, F(1,28) = 7.851; η^2^_partial_ = 0.22; p = 0.009, and main effect of adaptor was significant, F(1,28) = 19.715; η^2^_partial_ = 0.41; p<0.001. However the interaction between adaptor and experiment was non-significant, F(1,28) = 4.017; η^2^_partial_ = 0.13; p = 0.055. Hence our data did not provide sufficient evidence for the adaptation after-effects being modulated by different emotions.

If emotion adaptation is different from social context adaptation, we would expect the modulation of the adaptation effect to be different between experiment 1 and 3. We directly compared the adaptation modulation of experiment 1 and experiment 3 in a three way mixed ANOVA with adaptor as a within subject factor, experiment condition (the two adaptors were showing congruent vs. incongruent actions/emotions) as a between subject factor, and experiment as a between subject factor (experiment 1 vs experiment 3). A different modulation of the adaptation effect between experiment 1 and 3 would be indicated by a significant three way interaction between adaptor, experiment condition, and experiment. This three-way interaction suggest that the effect of congruency on the adaptation effect is different for experiment 1 and 3. For sake of clarity, we only report the significant effects of this three-way ANOVA (p≤0.05). We found a significant main effect of experiment condition, F(1,56) = 13.48, η^2^_partial_ = 0.20; p = 0.001, a significant effect of adaptor, F(1,56) = 13.79, η^2^_partial_ = 0.20; p<0.001, a significant interaction between adaptor and experiment condition, F(1,56) = 24.17, η^2^_partial_ = 0.30; p<0.001, and a significant three-way interaction between adaptor, experiment condition, and experiment, F(1,56) = 8.51, η^2^_partial_ = 0.13; p = 0.005. The three way interaction suggests a significant difference in the modulation of the adaptation effect by experimental condition between experiment 1 and 3.

Experiment 1 to 3 suggest that social context influences action recognition. Where within the action recognition hierarchy does this influence of social context emerge? The next experiment assessed whether visual information about an action is required to induce a social context modulation of the action adaptation effect. If visual information about an action is required for the social context modulation of the action adaptation effect, then social context sensitive visual action recognition processes are most likely affected by this modulation. Such a result would provide further evidence for the idea that *visual* action recognition processes are sensitive to social context.

To examine whether visual action information is required for the social context modulation of the action adaptation effect, experiment 4 assessed whether adaptation effects are found when participants adapted to the semantic action knowledge about an action. This semantic action knowledge was provided by the presentation of action words, which do not contain direct visual information about the action.

## Experiment 4

The social context modulation is likely to be mediated by high level processes in action recognition. One plausible explanation for the origin of adaptation effect modulation can be found in the adaptation of high level processes that are independent of the action’s physical visual appearance but sensitive to the action semantics. In experiment 4, we assessed the plausibility of this suggestion. We examined to what degree adaptation to linguistically induced semantic action knowledge induces action adaptation effects. To do so, we used the words ‘hitting’ and ‘waving’ as adaptors. If semantic action knowledge is not able to induce adaptation effects, processes mainly dedicated to the semantic action knowledge are unlikely to be at the heart of the social context modulation of the action adaptation effects.

### Methods

The methods were identical to experiment 3 with the following exceptions.

#### Participants

5 participants participated in the hitting context experiment (7 females, mean age = 29.67, SD = 7.78). All participants were naïve to the stimuli and the experimental procedure.

#### Stimuli and Apparatus

The two adaptor images were images of words written in white font on black background. Each adaptor displayed a different word (“hitting” or “waving”).

#### Procedure

Participants were asked to read the adaptation words repeatedly. The experiment took about 1.25 hours.

### Results

**Figure 6:**
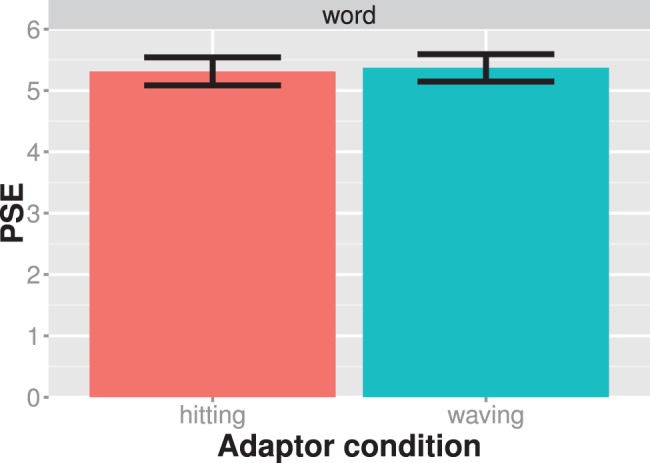
Results of experiment 4. The average PSE is shown for each adaptor condition separately. Larger PSE values indicate that the test stimulus showed more of a waving action. Bars always indicate one standard error of the mean.

The results of experiment 4 are shown in Figure 6. There is little difference between the two adaptor conditions. Our statistical analysis showed that the word adaptors did not induce a bias in the viewer’s perception of the test stimuli as measured by the shift of the psychometric functions at the PSE (t_paired_ = 1.642; df = 14; Cohen’s d = 0.42; p = 0.123). We therefore refrained from further examining the modulation of the adaptation effect by semantic adaptation. The lack of evidence for an action adaptation effect makes it unlikely that action adaptation is mediated by linguistically induced semantic knowledge about an action.

## General Discussion

We examined the influence of social context on action recognition using an action adaptation paradigm. In experiment 1, we found that social context preceding an action adaptor modulated the action adaptation after-effect. This modulation of the adaptation effect suggests that social context influences action recognition. In several experiments we examined the what degree this effect pertains to the adaptation of visual action recognition processes. The findings of experiment 2 highlighted the importance of the adaptation phase for the social context modulation of the adaptation effect. Without the presentation of the adaptor phase, adaptation effects were significantly weaker, and the social context modulation vanished. Additionally, we ruled out an obvious alternative explanation for the social context modulation of the adaptation effect, namely emotion adaptation (experiment 3). Because the social context manipulation also induced changes in the type of emotion displayed by the adaptors, we examined whether emotion adaptation played a confounding role in the modulation of the adaptation effect of experiment 1. Experiment 3 showed that the manipulation of emotional content was unable to induce a modulation of the adaptation effect similar to experiment 1. Specifically, we found the modulation effect in experiment 1 to be significantly larger than in experiment 3. These results run counter to the hypothesis that the modulation of the adaptation effect in experiment 1 was merely driven by the adaptation to the emotional content of the displayed actions. Finally, in experiment 4 we examined visual aspects of the visual action adaptation effect in experiment 1. We found no action adaptation effects if action information was only provided semantically through words, which makes it unlikely that non-visual semantic action processes are at the core of the social context modulation of the action adaptation effect.

The presentation of the social context alone seems to induce action adaptation effects as indicated by the significant main effect of (the video) adaptor in experiment 2. Consequently, it is likely that the video adaptor also contributed to the adaptation effect in experiment 1 thereby essentially prolonging the adaptation period of the action image adaptor. Longer the adaptation periods have been shown to increase the action adaptation effect ([18], [23]. On the other hand the inter-stimulus interval (ISI) between the video and adaptor (820 ms) most likely counteracted some of the contribution of the video. Barraclough and colleagues [18], [23] have also shown that adaptation effects decrease in a logarithmic fashion with increasing ISI. Hence the influence of the video on the adaptation period is most likely somewhat reduced due to the 820 ms ISI between the video and the static adaptor. The contribution of the video adaptor to action adaptation in experiment 1, however, does not alter the conclusion drawn from experiment 1: the critical contrast in experiment 1 consisted of comparing the hitting and waving context condition, which both showed a video adaptor to participants.

In addition, emotions might have contributed to the adaptation effect in experiment 1. Although the congruency of adaptor emotions were unable to modulate the action adaptation effect (experiment 3), the close-to-significant p-value (p = 0.055) indicates a tendency emotions to take an influence on action adaptation. We, therefore, do not want to exclude the possibility of an at least small effect of emotion on the action adaptation in experiment 1. However, the results of experiment 3 strongly suggest that the modulation of the adaptation effect is not driven by emotion alone.

Our adaptation effects can be distinguished from other forms of cognitive-perceptual bias, such as priming [25]. Priming refers to the altered participants response to a probe display after the presentation of another stimulus (prime). Positive priming effects are typically associated with a facilitatory effect of the prime display on the subsequent response to the probe display. This positive effect in contrast to the observed repelling (inhibitory) effects of the adaptor on the classification of the test stimulus. That is, participants are less likely to classify an ambiguous action as e.g. hitting after they have seen a hitting adaptor. Negative priming effects refer to an inhibited participant’s response to an object after the object had previously been ignored [26]. Negative priming is unlikely to account for the effects because we gave participants explicitly the instructions to look at the adaptors and not to ignore them. Reverse priming is another response bias that causes participants to show inhibited responses to a probe stimulus after a semantically similar stimulus had been previously shown. Reverse priming effects have been found in affective evaluation tasks and can be even induced linguistically using words [24], [27]. Due to the semantic linguistic nature of these reverse priming effects, one would expect to find reverse priming effects in Experiment 4, which used words as primes. However, incongruent with these reverse priming predictions, our results showed no change of response bias. In addition, reverse priming effects would be expected for much shorter adaptor presentation times (e.g. 200 ms). Experiment 2 showed action information much more briefly compared to experiment 1. However, the lack of an action adaptation modulation in experiment 2 are inconsistent with the predictions of a reverse priming effect. Because of these differences between predicted priming effects and actual observed effects, we think that it is unlikely that our results are owed to priming effects.

The results are interesting for the ongoing discussion about whether processes underlying visual action recognition encode action goals or primarily visual information about actions [8]. Experiment 4 suggest that non-visual action information is unlikely to cause a social context modulation of the action adaptation effect (experiment 4). This observation suggests that context sensitive visual action recognition processes mediate the social context modulation of the adaptation effect. The social context sensitivity of visual action recognition processes are in line with the idea that visual action recognition processes encode action goals. On the other hand, a wealth of previous research has demonstrated that action recognition mechanisms are also sensitive to low level features [28]. Moreover, physiologically plausible models of action recognition have demonstrated that many action recognition results can be explained with feed-forward processing of visual action information [5], [7], [29], [30]. In light of this, we therefore suggest that action recognition is sensitive to both action intentions and low level visual information.

The social context sensitivity of visual action recognition is likely to play an important role in human’s social functioning. By integrating current visual action information within a broader temporal-social context, humans might be able to disambiguate otherwise ambiguous action information. This processes would allow humans to accurately recognize the altered meaning of the same action in different social contexts.

The context sensitivity of action adaptation effects in visual recognition add to the recently emerging body of evidence that mechanisms underlying motor control [31], visual observation [10], imitation [32], and emotional bodily expression recognition [33] dependent on factors other than the immediate visual information pertaining to the body. It is also congruent with previous findings demonstrating the importance of the immediate action context on action discrimination performance, in particular, the ability of participants to tell individual and social interactions apart [34]–[37]. Our results extend previous empirical findings suggesting that temporally preceding social context is able to affect action recognition mechanisms.

This study is among the first to apply action adaptation paradigm to the categorization of different action types. Previous action adaptation studies [1], [18] investigated visual action recognition within the same locomotive (e.g. walking) and object-directed action (e.g. grasping) category. While these studies also have direct and important implications for categorization of different action types, we directly examined visual action recognition of different social action categories (e.g. handshake vs. waving) using an adaptation paradigm.

An interesting question for future research concerns the nature of the social context and the action information that is able to influence action recognition. To date, little known about this topic and, hence, we can only speculate about important factors. The influence of social context on the action adaptation effect is likely to be mediated by high level cognitive processes (e.g. attention). In addition, experiment 4 demonstrated that high level semantic action information alone is not sufficient for a social context modulation of action recognition effects. This observation points to the importance of visual action information in action recognition. In spite of this result, we do not want to exclude the possibility that non-visual information can also be effective in inducing a modulation of the adaptation effect. In experiment 4, participants did not deeply processed action information because they were told to read the word but not deeply encode it, e.g. by imagining the action. The deeper processing of action information, e.g. by means of imagination, could be critical to activate action recognition processes. Imagination has previously shown to illicit cortical responses that resemble actual visual stimulation [38]. As for action recognition, the imagination and observation of an action both activate the a cortical area considered critical for action recognition (dorsal premotor cortex) [39] although imagination and observation are overall associated with different cortical activation pattern [40]. Imagination seems to cause cortical activation patterns that at least partly resemble those of actual sensory stimulation. In this light, it seems possible that non-visual action information could also induce action adaptation effects if this information is processed more sufficiently deep by the observer, e.g. by means of imagining the action.

Here we showed that action adaptation after-effects are sensitive to the social context. These findings suggest that neural mechanisms involved in the visual recognition of an action are sensitive to social context information. Our results support the hypothesis that neural mechanisms contributing to visual action recognition are modulated by high-level properties of the stimulus such as its interpretation of an action and the viewer’s expectation about an action.
